# Ethnic Variations in Help-Seeking and Accessing Treatment for Eating Disorders: A Systematic Review of Qualitative Studies

**DOI:** 10.1192/bjo.2026.11226

**Published:** 2026-06-30

**Authors:** Melissa Chang, Manvir Dobb, Helena Tuomainen

**Affiliations:** University of Warwick, Coventry, United Kingdom

## Abstract

**Aims:**

Eating disorders (ED) are serious mental health conditions characterised by disordered eating behaviour and associated emotional distress. EDs are associated with high rates of mortality and morbidity, yet rates of help-seeking remain low. There is a misconception that EDs only affect “skinny, white, affluent girls” (SWAG) although anyone can be affected. Likewise, despite similar rates in ethnic minorities, studies suggest that help-seeking from this population remain low. The aim of this paper is to identify key barriers and facilitators to accessing ED treatment in the UK and whether ethnic minority groups have different experiences from the majority population in accessing treatment. We hypothesised that barriers and facilitators would differ between ethnic minority and majority populations in the UK.

**Methods:**

A systematic review of qualitative and mixed methods studies was conducted following PRISMA guidelines. PsycINFO, Embase, Medline and CINAHL databases were searched for relevant studies published since 2013. Two independent reviewers screened titles/abstracts and reviewed full texts. Eligible articles were coded in NVivo to generate themes following Braun and Clarke’s thematic synthesis guidelines.

**Results:**

Out of14 included studies, two focused exclusively on ethnic minority experiences. Across the studies, commonly shared barriers to accessing care included rigid ED guidelines, negative healthcare professional experiences, difficulty in self-recognising ED, low accessibility and awareness of services, and negative social relationships. Reported facilitators included improving education and recognition of EDs, informed and accessible delivery of care, and supportive communities. The two studies exploring the experiences of people of South Asian heritage highlighted additional barriers, such as concerns about doctor-patient confidentiality, and marriage-related pressures linked to beauty standards. Unique facilitators included suggestions for raising awareness of eating disorders through culturally relevant platforms.

**Conclusion:**

A more holistic approach is required from services and guidelines to tackle stigma associated with EDs and make services more accessible. There is a lack of primary research focusing on ethnic minorities, and further research is required.

